# Resident Memory T Cells and Their Effect on Cancer

**DOI:** 10.3390/vaccines8040562

**Published:** 2020-10-01

**Authors:** Daniel J. Craig, Justin F. Creeden, Katelyn R. Einloth, Cassidy E. Gillman, Laura Stanbery, Danae Hamouda, Gerald Edelman, Lance Dworkin, John J. Nemunaitis

**Affiliations:** 1 Department of Chemical Engineering, University of Toledo Medical Center, Toledo, OH 43614, USA; daniel.craig@rockets.utoledo.edu (D.J.C.); justin.creeden@rockets.utoledo.edu (J.F.C.); katelyn.einloth@rockets.utoledo.edu (K.R.E.); cassidy.gillman@rockets.utoledo.edu (C.E.G.); danae.hamouda@utoledo.edu (D.H.); gerald.edelman@utoledo.edu (G.E.); lance.dworkin@utoledo.edu (L.D.); 2 Gradalis, Inc., Carrollton, TX 75006, USA; lnejedlik@gradalisinc.com

**Keywords:** memory T cells, immunotherapy, cancer vaccine

## Abstract

Resident memory T (T_RM_) cells are a unique subset of CD8^+^ T cells that are present within certain tissues and do not recirculate through the blood. Long term memory establishment and maintenance are dependent on tissue population of memory T cells. They are characterized by dual CD69/CD103 positivity, and play a role in both response to viral infection and local cancer immunosurveillance. Human T_RM_ cells demonstrate the increased expression of adhesion molecules to facilitate tissue retention, have reduced proliferation and produce both regulatory and immune responsive cytokines. T_RM_ cell phenotype is often characterized by a distinct expression profile driven by Runx3, Blimp1, and Hobit transcription factors. The accumulation of T_RM_ cells in tumors is associated with increased survival and response to immunotherapies, including anti-PD-1 and anti-CTLA-4. In this review, we explore potential mechanisms of T_RM_ cell transformation and maintenance, as well as potential applications for the use of T_RM_ cells in both the development of supportive therapies and establishing more accurate prognoses.

## 1. Introduction

**Figure 1:**
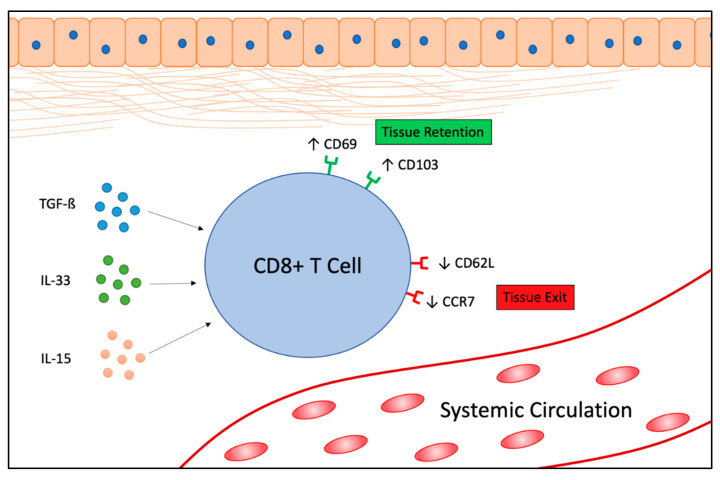
CD8^+^ T cells enter tissues and are stimulated by TGF-ß, IL-33, and IL-15 to upregulate the tissue-retention surface markers, CD69 and CD103, while simultaneously down-regulating the tissue-egress markers, CD62L and CCR7. This creates a resident memory T (T_RM_) cell phenotype, enabling T_RM_ cells to maintain close contact with malignant cells residing within tissues.

CD8^+^ T cells are a critical component of the adaptive immune system that target intracellular pathogens, such as virus-infected host cells, as well as host cells with oncogenic mutations. Based on the heterogeneity of cell-surface receptors isolated from peripheral blood samples, CD8^+^ T cells are classically stratified into two groups, effector memory T (T_EM_) cells or central memory T (T_CM_) cells [1]. Antigen presenting cells, such as dendritic cells, utilize the major histocompatibility complex I (MHC-I) system to present intracellular antigens to both recirculating and non-recirculating CD8^+^ T cells resulting in cell death [2]. More recent examination of nonlymphoid tissue specimens have revealed a unique population of non-recirculating T cells, called resident memory T (T_RM_) cells. T_RM_ cells do not re-circulate, and it is for this reason that these cells were not originally observed in samples obtained from peripheral blood [3]. Instead, T_RM_ cells reside in peripheral tissues long after an infection has cleared. After exposure to an antigen, T_RM_ cells persist, poised to respond quickly in the event of re-exposure by rapidly secreting cytotoxic granules as well as cytokines that recruit both innate and adaptive immune cells. This allows patients to respond to re-exposure much faster than T_EM_ or T_CM_ cells allow [4,5,6]. In addition to viral infections, T_RM_ cells are thought to take part in local cancer immunosurveillance because they often accumulate in a variety of human solid tumors, and are associated with positive patient outcomes [7,8,9,10,11]. This aligns with previous observations concerning the role that MHC-I antigen presentation systems play in detecting intracellular insults, such as viral infections and malignant transformation of host cells [12]. The T_RM_ cell phenotype is induced when effector-like CD8^+^ T cells—which enter a tissue early on in an infection—are activated by TGF-β, IL-33, and IL-15. Activation results in the expression of the transcription factors Hobit and Blimp1 [13]. This, in turn, initiates a signaling cascade that upregulates genes associated with tissue retention and downregulates genes associated with recirculation. The functional phenotype of CD8^+^ T_RM_ cells is largely due to their dual presentation of CD69 and CD103 surface markers, as well as an absence of the lymph node homing receptors CD62L and CCR7 (Figure 1) [7,14,15]. CD69 is a C-type lectin that is upregulated prior to CD103 and antagonizes sphingosine-1-phosphate-receptor-1 (S1P1), rendering T_RM_ cells unresponsive to tissue egress signals and resulting in tissue retention [15,16]. CD103 is an αEβ7 integrin that binds to the epithelial marker E-cadherin, which allows T_RM_ cells to maintain close contact with both peripheral lymphoid and non-lymphoid tissues, such as the lung, skin, gastrointestinal, and genitourinary tracts [17]. CD103-mediated binding to E-cadherin also allows T_RM_ cells to maintain close contact with malignant cells residing within these tissues [18,19]. This allows T_RM_ cells to act as an immunosurveillance population within the tissue microenvironment (TME). T_RM_ cells are primed with both cytotoxic granules and effector molecules, such as IFN-γ and TNF-α, which can further activate additional immune cells [4,20]. The differentiation and maintenance of T_RM_ cells also requires Runx3 expression, which serves as a master regulator of T_RM_ cell phenotype by regulating expression of CD103 and CD69, and increasing cytotoxic activity [21,22]. This is evident in preclinical murine melanoma models, where *Runx3*-shRNAmir knockdown resulted in diminished T_RM_ cell accumulation within tumors, uncontrolled tumor growth, and low survival. Conversely, T_RM_ cells that overexpress Runx3 resulted in growth inhibition and improved survival [21].

## 2. T_RM_ Cells in Cancer

### 2.1. Function of T_RM_ Cells in Cancer

The presence of T_RM_ cells in clinical tumor samples is associated with an improved outcome in a variety of cancers, including melanoma [23], non-small cell lung cancer (NSCLC) [9], breast cancer [24,25,26], cervical cancer [11], and ovarian cancer [27]. In addition, T_RM_ cells confer a degree of antitumor immunity in murine melanoma models. Specifically, knockout mice lacking CD69 or CD103 were more susceptible to transplantable melanoma challenge relative to wild-type mice [28,29]. Furthermore, T_RM_ cells pre-generated using vaccines containing tumor neoantigen DNA are protective against transplantable melanoma or head and neck tumors even when circulating T cells are depleted, using targeting antibodies [30,31]. Mackay et al. demonstrated that local signaling by IL-15 and TGF-β were required for T_RM_ cell transformation and the maintenance of CD69 and CD103 status in skin epithelium [15]. T_RM_ cells utilize a variety of tumor-killing methods depending on the cancer type. For example, samples from NSCLC patients demonstrate marked antigen-independent increases in inflammatory cytokine mRNA expression of granzyme B, IFN-γ, and TNF-α [32], providing a potential explanation for the ability of T_RM_ cells to act quickly following antigen exposure. Tumor samples from urothelial urinary bladder cancer (UBC) patients were obtained during diagnostic transurethral resections, and T_RM_ cells were isolated using flow cytometry. The pyrosequencing of genes associated with cytotoxicity reveal that the perforin gene (PRF1) is hypomethylated in T_RM_ cells present in UBC samples. This corresponds to the increased perforin expression previously reported in T_RM_ cells from other cancers [32,33]. In addition, T_RM_ cells derived from a variety of primary ovarian tumors, including endometrioid, mucinous, clear cell, and high-grade serous carcinoma, were obtained and sorted using flow cytometry and immunohistochemistry. The authors found that the T_RM_ cells universally expressed high amounts of TIA-1, a marker of cytotoxic potential, relative to non-resident CD103^−^ T cells [34]. The number and frequency of T_RM_ cells vary from cancer to cancer, patient to patient, and even from lesion to lesion within the same patient [23,35,36]. This suggests that environmental cues from the tumor microenvironment are essential for the recruitment and maintenance of T_RM_ cells. Metabolically active tumor cells are heavily dependent on glucose metabolism, commonly referred to as the “Warburg effect,” resulting in elevated lactate levels in the tumor microenvironment [37]. This setting of nutrient deprivation and local acidosis favors metastasis, angiogenesis, and immunosuppression. T_RM_ cells have a unique ability to adapt to this by metabolizing free fatty acids. This suggests that these cells are better suited to survive in the TME [38].

In addition to predicting survival, the induction or presence of T_RM_ cells enhance response to certain therapeutics. T_RM_ cells are often present in normal tissues and tumors which express higher amounts of immune inhibitory and costimulatory receptors, such as PD-1, CTLA-4, and Tim3. This prevents autoimmune reactions [15,32,39]. Interestingly, the combination of immune inhibitory and costimulatory receptors varies depending on tumor type [27]. This opens the door for immunotherapies that seek to target these receptors and enhance T cell response. Enamorado et al. demonstrate this in a preclinical murine model in which anti-PD-1 antibody administration and adoptive T_RM_ cell transfer inhibit the growth of subcutaneously injected MC38-OVA tumors, as well as intradermal B16-OVA tumors, when compared to adoptive T_RM_ cell therapy alone [40]. Edwards et al. isolated T_RM_ cells from both immunotherapy-naïve melanoma samples and those derived from patients undergoing anti-PD-1 therapy using multiparameter flow cytometry. T_RM_ cells were quantified using quantitative multiplex immunofluorescence staining to show that the presence of T_RM_ cells in immunotherapy-naïve melanoma samples was associated with significantly increased melanoma-specific survival, and this cell population was further expanded using anti-PD-1 therapy [23]. Additionally, Blanc et al. showed that the T_RM_ cell population increases within tumors during the early stages of anti-PD-1 treatment [41]. In addition, T_RM_ cells isolated from lung carcinomas co-cultured with autologous tumor cells demonstrate enhanced cytotoxic activity in the presence of PD-1 targeting antibodies compared to those not treated with PD-1 antibodies [9]. Also, the combination of PD-1 targeting antibodies with PPAR-α agonists or the administration of free fatty acids increased functionality of T_RM_ cells in a melanoma model [42]. These studies provide evidence for the superior prognostic value of the number of T_RM_ cells present in multiple tumor types compared to circulating CD8^+^ cells. This evidence demonstrates that T_RM_ cells are not only associated with protective immunity, but they may also be effective in increasing the response to anti-PD-1 therapy [23,43].

### 2.2. Identification of T_RM_ Cells in Patient Samples

T_RM_ cells have been identified in both human and murine tissues, including liver, lungs, pancreas, lymphoid tissues, genital mucosa, stomach, jejunum, ileum, colon, bone marrow, and in brain obtained from autopsies [44,45,46,47,48,49,50,51]. However, since T_RM_ cells do not recirculate in blood, point-of-care collection and analysis of T_RM_ cells is limited by the need for tissue biopsy or surgical resection [44]. Once appropriate samples are obtained, there are a variety of methods for characterizing T_RM_ cells. For example, the cell surface epitopes CD103 and CD69 can be used to separate T_RM_ cells from other CD8^+^ T cells using flow cytometry. T_RM_ cells can also be identified through the use of unique transcriptional signatures that include the increased expression of IL-2, IFN-γ, IL-17, and IL-10, as well as multiplex immunohistochemistry [34,39]. In addition, T_RM_ cells can be differentiated from circulating T cells via functional characterization by assessing T_RM_ disequilibrium in a parabiosis model [52,53,54]. Another method of identifying T_RM_ cells is by their migration patterns using methods such as photoreaction, as seen in transgenic murine and lymphatic cannulation models [53,55,56,57]. The accurate and quantitative measurement of T_RM_ will facilitate potential use as a biomarker involved in clinical testing of immunotherapy.

### 2.3. Improving Vaccine Efficacy

The human papilloma virus (HPV) cancer vaccine provides long-term protection against certain cancers associated with HPV, including cervical cancer, as well as head and neck cancer. The success of these vaccines is likely associated with the presence of T_RM_ cells which are knowledgeable of the HPV-induced cancer antigen profile [58]. Despite the fact that HPV-induced cancers primarily develop at mucosal sites such as the oral and vaginal cavities, the majority of preclinical vaccines are administered subcutaneously or intramuscularly [59]. In murine models, intravaginal boosters, following systemic (intramuscular) vaccination, resulted in the local accumulation of T_RM_ cells and was associated with increased survival compared to intramuscular vaccination alone, which did not induce local T_RM_ cell accumulation [60]. In addition, intranasal administration of the HPV vaccine in an orthotopic head and neck cancer model provided long-lasting protection by recruiting and maintaining T_RM_ cells in the local tissue. Intranasal vaccination for respiratory syncytial virus (RSV) also generated robust and durable T_RM_ cell populations that were not detectable after subcutaneous vaccination [61].

Vaccine trials involving intramuscular injections for herpes simplex virus 2 (HSV-2) have largely been ineffective. This is likely due to the minimal recruitment of T_RM_ cells to the site of infection. To address this limitation, Cuburu et al. generated a replication-defective HPV pseudovirus that expressed HSV-2 glycoproteins B (gB) and D (gD), for intravaginal vaccination using a murine model. Mice vaccinated intravaginally demonstrated a significantly reduced viral load and reduced severity of HSV lesions compared to intramuscular vaccination. Importantly, intravaginal vaccination resulted in the accumulation of T_RM_ cells that were able to secrete IFN-γ, TNF-α, and moderate levels of neutralizing antibodies [62].

This phenomenon is further supported by preclinical glioblastoma models, whereby the injection of tumor cells by different routes (intraperitoneal, intracranial, and subcutaneous) resulted in T cells displaying different patterns of integrins depending on the sentinel lymph nodes they were obtained from. This suggests that the route of immunization plays an important role in vaccine efficacy [63,64]. Importantly, the use of a potent circulating memory T cell inhibitor (FTY720) provided evidence that the T_RM_ cells alone could partially control tumor growth, although the presence of circulating memory T cells improved vaccine efficacy [30,65]. However, while the interplay between both T_RM_ cells and circulating memory T cells is critical to vaccine efficacy, the presence of local T_RM_ cells appears to be paramount [40].

Reports describing the Vigil vaccine in various solid tumors provide additional evidence supporting the efficacy of immunization. Vigil is an autologous vaccine produced from harvested tumor tissue and transfected ex vivo, using a plasmid containing the GM-CSF gene and short hairpin RNA that knocks down furin expression [66]. Furin is a convertase which is responsible for the cleavage and activation of TGFβ1 and TGFβ2. Phase I clinical trials in Ewings sarcoma, melanoma, and solid tumor malignancies demonstrate both safety and efficacy [67,68,69,70]. A phase I trial investigating the Vigil vaccine in solid tumor patients reports a significant correlation between γ-IFN-ELISPOT positive response and improved overall survival [69,70]. Subsequently, a Phase IIa trial of Vigil in ovarian cancer patients demonstrated safety and improved relapse-free survival compared to control [71]. A Phase IIb trial has recently completed and significant survival advantage in relapse free survival (RFS) was demonstrated in patients with *BRCA*-wt tumors [72]. Based on the durability of clinical response observed in Phase I testing and long term follow up, it was suggested that, Vigil induces persistent circulating “self” mononuclear cell function activity against “self” tumor following treatment and persists after discontinuation. Evidence supports enhanced memory T cell function which maybe relevant to clonal neoantigens [70,71,73]. Further study is indicated.

### 2.4. Improving Adoptive T Cell Therapy

In addition to cancer vaccines, direct infusion of cancer neoantigen experienced T_RM_ cells through adoptive T cell therapy (ACT) is a promising strategy. Traditional ACT involves harvesting tumor-specific circulating T cells and expanding them exponentially in vitro. These T cells are then reinfused into the patient, where they mediate tumor destruction [74]. Milner et al. provided evidence that ACT with T_RM_ cells inhibited tumor growth and increased overall survival in mice [21]. Using a murine model for adoptive T cell therapy in melanoma, they demonstrated that transfer of Runx3-deficient CD8^+^ T cells (recirculating phenotype) resulted in increased mortality due to their inability to accumulate in tumors. Conversely, the transfer of CD8^+^ T cells overexpressing Runx3 (T_RM_ cell phenotype) resulted in an accumulation of T_RM_ cells in tumors and prolonged survival [21]. In addition, reprogramming tumor infiltrating dendritic cells using β-glucan curdlan resulted in increased dendritic cell TGF-β production and the differentiation of CD103^+^ T cells in a humanized murine model of breast cancer. Importantly, this resulted in tumor rejection, highlighting how indirect adoptive cell therapy may lead to local T_RM_ cell transformation and maintenance [75].

A more recent branch of adoptive cell transfer has been developed, in which a patient’s T cells are collected and modified to produce and present chimeric antigen receptors (CARs) on their surface. These receptors enable the new CAR T-cells to latch onto tumor-specific antigens on the cell’s surface. Currently, CAR-T therapy is used to treat chemotherapy-resistant acute lymphoblastic leukemia (ALL), chronic lymphocytic leukemia (CLL), and non-Hodgkin lymphoma (NHL) [76,77,78,79,80]. Unfortunately, attempts to use CAR-T therapy to treat solid tumors have not been nearly as successful [81]. At least part of the difficulty in treating solid tumors is ensuring that the CAR-T cells reach and infiltrate the tumor sites [82]. Although the precise mechanism of differentiation and maintenance of T_RM_ cells are still being elucidated, researchers may be able to use CAR-T therapy to increase and maintain functional T_RM_ cells in cancer patients with solid tumors by inducing the T_RM_ cell phenotype with TGF-β and IL-15, or via other relevant molecular profile induction [83].

### 2.5. T_RM_ Cell Clinical Trials

Few clinical trials have explored how the presence of T_RM_ cells in various cancers are related to response rates with immune checkpoint inhibitors (Table 1). However, Savas et al. demonstrated the importance of both the CD8^+^/CD103^+^ and CD8^+^/CD103^−^ T cell subtypes in prolonging overall survival in triple-negative and HER2-positive breast cancer using single-cell profiling (*p* = 0.03) [26]. The authors found a 37-gene T_RM_ signature associated with better response to cancer treatments, using both multi-cell RNA-Seq of CD8^+^/CD103^+^ T cells (T_RM_ phenotype) compared to CD8^+^/CD103^−^ T cells, as well as single-cell RNA-Seq [26]. Among the 37 genes included in the gene signature, there was a significant decrease in the tissue-egress genes, S1PR1 and KLF2, and a significant increase in expression of immune checkpoint genes, PD1 and CTLA-4, in CD8^+^/CD103^+^ T cells, compared to CD8^+^/CD103^−^ T cells, suggesting a functionally and phenotypically distinct subset of CD8^+^ T cells.

The presence of CD103+ TILs was also evaluated in a cohort of breast cancer cases from the Manitoba Breast Tumor Bank. CD103 TILs in the intraepithelial compartment correlated with increased relapse free and overall survival (OS) in basal-like tumors (HR 0.28; CI 0.17–0.72, *p* = 0.0047 and HR 0.25; CI 0.17–0.66 *p* = 0.0017 respectively). Increased levels of CD8^+^ /CD103^+^ TILs were also strongly associated with increased clinical benefit in both RFS and OS (HR 0.10 CI 0.07–0.62, *p* = 0.006 and HR 0.09 CI 0.07–0.57, *p* = 0.003 respectively) [8]. These results further confirm the importance of CD8^+^/CD103^+^ TILs in breast cancer.

The I-SPY 2 trial (NCT01042379) is an ongoing neoadjuvant platform trial evaluating the efficacy of a variety of experimental agents/combinations when added to standard chemotherapy to treat multiple breast cancer types. The goal of this study is to identify molecular signatures that serve as early indicators of treatment success. The trial is open to all women 18 and over with radiologically diagnosed stage II-III breast malignancies who have not received any prior cytotoxic treatment. Patients are enrolled and assessed for HER2, HR, and MammaPrint status, using IHC, FISH, and HER2 expression, generating eight defined subgroups based on biomarker status. Yau et al. evaluated a subset of this data to compare the prognostic value of published T/B cell signatures including subsets exhibiting a CD8^+^ T resident memory (T_RM_) phenotype or CD8^+^ T effector memory (T_EM_) phenotype. Using gene expression data from pretreatment biopsies of 989 patients enrolled in I-SPY 2 and logistic modeling to predict pathologic complete response (pCR), they found that the T_RM_ cell phenotype was most predictive of survival in the HR^—^/HER2^—^ subtype [88].

The Keynote-086 trial (NCT02447003) is a recently published international, open-label, multicohort, phase II clinical trial exploring the efficacy of the anti-PD-1 antibody pembrolizumab (MK-3475) in the treatment of metastatic TNBC. This study enrolled patients in two cohorts. Cohort A consisted of 170 patients with recurrent disease irrespective of PD-L1 expression status, who received at least 1 line of prior therapy that did not include: an anti-PD1, anti-PD-L1, anti-PD-L2, or another co-inhibitory T-cell receptor therapy at any time; an antineoplastic monoclonal antibody within four weeks; chemotherapy, targeted small molecule therapy, or radiation therapy within two weeks. Patients submitted a tumor biopsy sample that was evaluated for TNBC status and determination of PD-L1 status using gene expression measurement. Patients received 200 mg intravenous pembrolizumab every three weeks for up to two years until evidence of disease progression through radiologic confirmation, adverse effects or patient withdrawal. The overall response rate was 5.3% (95% CI 2.7–9.9), with 5.7% (95% CI 2.4–12.2) in the PD-L1-positive population, and 4.7% (95% CI 1.1–13.4) in the PD-L1 negative population. Median progression free survival was 2.0 months (95% CI 1.9–2.0) and median overall survival was 9.0 months (95% CI 7.6–11.2) [89]. Cohort B enrolled 84 patients with no prior lines of systemic treatment who were PD-L1 positive using gene expression. Patients received 200 mg intravenous pembrolizumab every three weeks for up to two years, until evidence of disease progression through radiologic confirmation, adverse effects or patient withdrawal. The overall response rate was 21.4% (95% CI 13.9–31.4), with 4 patients receiving a complete response and 14 exhibiting a partial response. Median progression free survival was 2.1 months (95% CI 2.0–2.2) and median overall survival was 18.0 months (95% CI, 12.9–23.0) [90]. Loi et al. examined Phase II of the study, which expanded the investigation into the efficacy of pembrolizumab to subgroups of patients from Phase I, including patients who demonstrated a T_RM_ gene signature based on RNA-seq analysis from their biopsy specimens. The authors found that expression of the T_RM_ signature was associated with increased PFS (*p* < 0.001) and OS (*p* < 0.001) in patients with advanced-stage TNBC treated with pembrolizumab monotherapy [91]. Several ongoing studies are evaluating the use of gene expression profiles to predict improved clinical outcomes (NCT02841748 and NCT03516981).

## 3. Future Directions

Checkpoint inhibitors have demonstrated remarkable clinical efficacy to achieve durable responses, however, a subset of patients do not respond to checkpoint inhibition, despite having an immunologically “hot” tumor, including PD-L1 expression, high TMB or other prognostic factors [92]. While clinical trial data is limited, generation of T_RM_ cells has shown prognostic value to determine which patients will have the most clinical benefit to checkpoint inhibitors. Moreover, the generation of T_RM_ cells through vaccination may provide a mechanism to sensitize tumors to check point inhibition. Ovarian cancer in particular has not responded to checkpoint inhibition, despite early trials indicating a favorable immune profile [93,94,95]. There have been clinical trials involving combination vaccination and checkpoint inhibitors, with most administering the combination concurrently. In melanoma, gp100, a glycoprotein peptide vaccine, was administered concurrently with ipilimumab, and no clinical benefit was seen with the combination compared to ipilimumab alone [96]. However, in a murine preclinical model of prostate cancer, mice who received GVAX prior to anti-CTLA-4 demonstrated increased CD8^+^ and CD4^+^ T cells compared to those who received anti-CTLA-4 treatment first [97]. These findings may indicate that the timing of combination vaccination and checkpoint inhibitor is critical to achieve response. This may be due to the ability of vaccination to prime T cells and promote the generation of T_RM_ prior to checkpoint inhibition which would rapidly expand this population. Further research is needed to determine if this approach can be used to sensitize patients to checkpoint inhibition.

TILs could also be used to identify a sensitive patient population prior to checkpoint therapy. The GeparNuevo phase II double-blind study in TNBC investigated the safety and efficacy of durvalumab stratified patients, based on stromal tumor infiltrating (sTILs) lymphocytes. Pathological complete response (pCR) was increased in patients with higher sTILs compared to low sTILs, indicating that the presence of sTILs prior to therapy start may serve as a prognostic marker [98]. Further analysis is ongoing to characterize the sTILs to determine the subpopulation molecular characteristics. It would be beneficial to determine the population of T_RM_ and how they relate to response and clinical outcome. More research is needed to determine if sTILs are a prognostic factor in other cancer types and with other checkpoint inhibitors.

The drawback to both of these methods would be the necessity of the tissue. These approaches would be limited to solid tumors with easily assessable surgical or biopsy sites, to obtain adequate tissue specimens in addition to those used for standard of care practices. In the case of vaccination, autologous vaccines require sufficient tissue to construct the vaccine, which then must be manufactured.

## 4. Conclusions

While much has been uncovered regarding the mechanism of T_RM_ cell transformation and maintenance, there are still missing pieces to the puzzle. Further evaluation of the molecular signal patterns may provide direction to additional therapies. In addition to developing supportive regimens, the further characterization of T_RM_ cell status in clinically derived patient samples may also be used as a biomarker to predict response to therapy and prognosis, particularly immunotherapies involving checkpoint inhibitors, CAR-T approaches and/or vaccines, such as those with multiple immune stimulatory functions (i.e., Vigil).

## Figures and Tables

**Table 1:** Studies in solid tumors evaluating T_RM_ cell populations reporting clinical benefit.

Tumor Type	T_RM_ Markers	No. of Samples/Patients	Reference
**Ovarian Cancer**	CD103	489	[27]
	CD103	497	[34]
	CD103, CD3, TCRαβ, CD8αβ, CD4	186	[84]
**Cervical Cancer**	CD103	460	[11]
**Melanoma**	CD69, CD103, TNFRSF18, CD8	44	[23]
	CD8, CD103, CD69	18	[85]
**Lung Cancers**	CD8, CD103, CD3	101	[9]
	CD8, CD103	77	[10]
	CD8, CD103	510	[86]
**Pancreatic Cancer**	CD8, CD103	136	[87]
**Breast Cancer**	CD8, CD103	424	[8]
	CD8, T cell gene signatures	989	[88]

## References

[1] Sallusto F., Lenig D., Förster R., Lipp M., Lanzavecchia A. (1999). Two subsets of memory T lymphocytes with distinct homing potentials and effector functions. Nature.

[2] Fu C., Jiang A. (2018). Dendritic Cells and CD8 T Cell Immunity in Tumor Microenvironment. Front. Immunol..

[3] Masopust D., Thorpe S.J., Fabre-Thorpe M. (2001). Preferential Localization of Effector Memory Cells in Nonlymphoid Tissue. Science.

[4] Ariotti S., Hogenbirk M.A., Dijkgraaf F.E., Visser L.L., Hoekstra M.E., Song J.-Y., Jacobs H., Haanen J.B., Schumacher T.N. (2014). Skin-resident memory CD8+ T cells trigger a state of tissue-wide pathogen alert. Science.

[5] Mueller S.N., Gebhardt T., Carbone F.R., Heath W.R. (2013). Memory T Cell Subsets, Migration Patterns, and Tissue Residence. Annu. Rev. Immunol..

[6] Schenkel J.M., Fraser K.A., Beura L.K., Pauken K.E., Vezys V., Masopust D. (2014). Resident memory CD8 T cells trigger protective innate and adaptive immune responses. Science.

[7] Park S.L., Gebhardt T., Mackay L.K. (2019). Tissue-Resident Memory T Cells in Cancer Immunosurveillance. Trends Immunol..

[8] Wang Z.-Q., Milne K., DeRocher H., Webb J.R., Nelson B.H., Watson P.H. (2016). CD103 and Intratumoral Immune Response in Breast Cancer. Clin. Cancer Res..

[9] Djenidi F., Adam J., Goubar A., Durgeau A., Meurice G., De Montpreville V., Validire P., Besse B., Mami-Chouaib F. (2015). CD8+CD103+ Tumor–Infiltrating Lymphocytes Are Tumor-Specific Tissue-Resident Memory T Cells and a Prognostic Factor for Survival in Lung Cancer Patients. J. Immunol..

[10] Ganesan A.-P., Clarke J., Wood O., Garrido-Martin E.M., Chee S.J., Mellows T., Samaniego-Castruita D., Singh D., Seumois G., Alzetani A. (2017). Tissue-resident memory features are linked to the magnitude of cytotoxic T cell responses in human lung cancer. Nat. Immunol..

[11] Komdeur F.L., Prins T.M., Van De Wall S., Plat A., Wisman G.B.A., Hollema H., Daemen T., Church D.N., De Bruyn M., Nijman H.W. (2017). CD103+ tumor-infiltrating lymphocytes are tumor-reactive intraepithelial CD8+ T cells associated with prognostic benefit and therapy response in cervical cancer. OncoImmunology.

[12] Comber J.D., Philip R. (2014). MHC class I antigen presentation and implications for developing a new generation of therapeutic vaccines. Ther. Adv. Vaccines.

[13] Mackay L.K., Minnich M., Kragten N.A.M., Liao Y., Nota B., Seillet C., Zaid A., Man K., Preston S., Freestone D. (2016). Hobit and Blimp1 instruct a universal transcriptional program of tissue residency in lymphocytes. Science.

[14] Dumauthioz N., Labiano S., Romero P. (2018). Tumor Resident Memory T Cells: New Players in Immune Surveillance and Therapy. Front. Immunol..

[15] Mackay L.K., Rahimpour A., Ma J.Z., Collins N., Stock A.T., Hafon M.-L., Vega-Ramos J., Lauzurica P., Mueller S.N., Stefanovic T. (2013). The developmental pathway for CD103+CD8+ tissue-resident memory T cells of skin. Nat. Immunol..

[16] Mackay L.K., Braun A., MacLeod B.L., Collins N., Tebartz C., Bedoui S., Carbone F.R., Gebhardt T. (2015). Cutting Edge: CD69 Interference with Sphingosine-1-Phosphate Receptor Function Regulates Peripheral T Cell Retention. J. Immunol..

[17] Cepek K.L., Shaw S.K., Parker C.M., Russell G.J., Morrow J.S., Rimm D.L., Brenner M.B. (1994). Adhesion between epithelial cells and T lymphocytes mediated by E-cadherin and the αEβ7 integrin. Nature.

[18] Mueller S.N., Mackay L.K. (2015). Tissue-resident memory T cells: Local specialists in immune defence. Nat. Rev. Immunol..

[19] Mami-Chouaib F., Blanc C., Corgnac S., Hans S., Malenica I., Granier C., Tihy I., Tartour E. (2018). Resident memory T cells, critical components in tumor immunology. J. Immunother. Cancer.

[20] Schenkel J.M., Fraser K.A., Vezys V., Masopust D. (2013). Sensing and alarm function of resident memory CD8+ T cells. Nat. Immunol..

[21] Milner J.J., Toma C., Yu B., Zhang K., Omilusik K., Phan A.T., Wang D., Getzler A., Nguyen T., Crotty S. (2017). Runx3 programs CD8+ T cell residency in non-lymphoid tissues and tumours. Nature.

[22] Cruz-Guilloty F., Pipkin M.E., Djuretic I.M., Levanon D., Lotem J., Lichtenheld M.G., Groner Y., Rao A. (2009). Runx3 and T-box proteins cooperate to establish the transcriptional program of effector CTLs. J. Exp. Med..

[23] Edwards J., Wilmott J.S., Madore J., Gide T.N., Quek C.Y.J., Tasker A., Ferguson A.L., Chen J.-B., Hewavisenti R., Hersey P. (2018). CD103+ Tumor-Resident CD8+ T Cells Are Associated with Improved Survival in Immunotherapy-Naïve Melanoma Patients and Expand Significantly During Anti-PD-1 Treatment. Clin. Cancer Res..

[24] Adams S., Gray R.J., DeMaria S., Goldstein L., Perez E.A., Shulman L.N., Martino S., Wang M., Jones V.E., Saphner T.J. (2014). Prognostic Value of Tumor-Infiltrating Lymphocytes in Triple-Negative Breast Cancers From Two Phase III Randomized Adjuvant Breast Cancer Trials: ECOG 2197 and ECOG 1199. J. Clin. Oncol..

[25] Egelston C.A., Avalos C., Tu T.Y., Rosario A., Wang R., Solomon S., Srinivasan G., Nelson M.S., Huang Y., Lim M.H. (2019). Resident memory CD8+ T cells within cancer islands mediate survival in breast cancer patients. JCI Insight.

[26] Savas P., Virassamy B., Ye C., Salim A., Mintoff C.P., Caramia F., Salgado R., Byrne D.J., Teo Z.L., Kathleen Cuningham Foundation Consortium for research into Familial Breast cancer (kConFab) (2018). Single-cell profiling of breast cancer T cells reveals a tissue-resident memory subset associated with improved prognosis. Nat. Med..

[27] Webb J.R., Milne K., Nelson B.H. (2015). PD-1 and CD103 are widely co-expressed on prognostically favorable intraepithelial CD8 T cells in human ovarian cancer. Cancer Immunol. Res..

[28] Malik B.T., Byrne K.T., Vella J.L., Zhang P., Shabaneh T.B., Steinberg S.M., Molodtsov A.K., Bowers J.S., Angeles C.V., Paulos C.M. (2017). Resident memory T cells in the skin mediate durable immunity to melanoma. Sci. Immunol..

[29] Park S.L., Buzzai A., Rautela J., Hor J.L., Hochheiser K., Effern M., McBain N., Wagner T., Edwards J., McConville R. (2018). Tissue-resident memory CD8+ T cells promote melanoma–immune equilibrium in skin. Nature.

[30] Nizard M., Roussel H., Diniz M.O., Karaki S., Tran T., Voron T., Dransart E., Sandoval F., Riquet M., Rance B. (2017). Induction of resident memory T cells enhances the efficacy of cancer vaccine. Nat. Commun..

[31] Gálvez-Cancino F., López E., Menares E., Díaz X., Flores C., Cáceres P., Hidalgo S., Chovar O., Alcántara-Hernández M., Borgna V. (2018). Vaccination-induced skin-resident memory CD8+T cells mediate strong protection against cutaneous melanoma. OncoImmunology.

[32] Hombrink P., Helbig C.A., Backer R., Piet B.E., Oja A., Stark R., Brasser G., Jongejan A.E., Jonkers R., Nota B. (2016). Programs for the persistence, vigilance and control of human CD8+ lung-resident memory T cells. Nat. Immunol..

[33] Hartana C.A., Bergman E.A., Broomé A., Berglund S., Johansson M., Alamdari F.I., Jakubczyk T., Huge Y., Aljabery F., Palmqvist K. (2018). Tissue-resident memory T cells are epigenetically cytotoxic with signs of exhaustion in human urinary bladder cancer. Clin. Exp. Immunol..

[34] Webb J.R., Milne K., Watson P., DeLeeuw R.J., Nelson B.H. (2013). Tumor-Infiltrating Lymphocytes Expressing the Tissue Resident Memory Marker CD103 Are Associated with Increased Survival in High-Grade Serous Ovarian Cancer. Clin. Cancer Res..

[35] Simoni Y., Becht E., Fehlings M.G., Loh C.Y., Koo S.L., Teng K.W.W., Yeong J., Nahar R., Zhang T., Kared H. (2018). Bystander CD8+ T cells are abundant and phenotypically distinct in human tumour infiltrates. Nature.

[36] Boddupalli C.S., Bar N., Kadaveru K., Krauthammer M., Pornputtapong N., Mai Z., Ariyan S., Narayan D., Kluger H., Deng Y. (2016). Interlesional diversity of T cell receptors in melanoma with immune checkpoints enriched in tissue-resident memory T cells. JCI Insight.

[37] De La Cruz-López K.G., Castro-Muñoz L.J., Reyes-Hernández D.O., García-Carrancá A., Manzo-Merino J. (2019). Lactate in the Regulation of Tumor Microenvironment and Therapeutic Approaches. Front. Oncol..

[38] Pan Y., Tian T., Park C.O., Lofftus S.Y., Mei S., Liu X., Luo C., O’Malley J.T., Gehad A., Teague J.E. (2017). Survival of tissue-resident memory T cells requires exogenous lipid uptake and metabolism. Nature.

[39] Kumar B.V., Ma W., Miron M., Granot T., Guyer R.S., Carpenter D.J., Senda T., Sun X., Ho S.-H., Lerner H. (2017). Human Tissue-Resident Memory T Cells Are Defined by Core Transcriptional and Functional Signatures in Lymphoid and Mucosal Sites. Cell Rep..

[40] Enamorado M., Iborra S., Priego E., Cueto F.J., Quintana J.A., Martínez-Cano S., Mejías-Pérez E., Esteban M., Melero I., Hidalgo A. (2017). Enhanced anti-tumour immunity requires the interplay between resident and circulating memory CD8+ T cells. Nat. Commun..

[41] Blanc C., Hans S., Tran T., Granier C., Saldman A., Anson M., Oudard S., Tartour E. (2018). Targeting Resident Memory T Cells for Cancer Immunotherapy. Front. Immunol..

[42] Zhang Y., Kurupati R., Liu L., Zhou X.Y., Zhang G., Hudaihed A., Filisio F., Giles-Davis W., Xu X., Karakousis G.C. (2017). Enhancing CD8+ T Cell Fatty Acid Catabolism within a Metabolically Challenging Tumor Microenvironment Increases the Efficacy of Melanoma Immunotherapy. Cancer Cell.

[43] Wei S., Levine J.H., Cogdill A.P., Zhao Y., Anang N.-A., Andrews M.C., Sharma P., Wang J., Wargo J.A., Pe’Er D. (2017). Distinct Cellular Mechanisms Underlie Anti-CTLA-4 and Anti-PD-1 Checkpoint Blockade. Cell.

[44] Szabó P.A., Miron M., Farber D.L. (2019). Location, location, location: Tissue resident memory T cells in mice and humans. Sci. Immunol..

[45] Booth J.S., Toapanta F.R., Salerno-Goncalves R., Patil S., Kader H.A., Safta A.M., Czinn S.J., Greenwald B.D., Sztein M.B. (2014). Characterization and Functional Properties of Gastric Tissue-Resident Memory T Cells from Children, Adults, and the Elderly. Front. Immunol..

[46] Okhrimenko A., Grün J.R., Westendorf K., Fang Z., Reinke S., Von Roth P., Wassilew G., Kühl A.A., Kudernatsch R., Demski S. (2014). Human memory T cells from the bone marrow are resting and maintain long-lasting systemic memory. Proc. Natl. Acad. Sci. USA.

[47] Pallett L.J., Davies J., Colbeck E.J., Robertson F.P., Hansi N., Easom N.J., Burton A.R., Stegmann K.A., Schurich A., Swadling L. (2017). IL-2high tissue-resident T cells in the human liver: Sentinels for hepatotropic infection. J. Exp. Med..

[48] Wong M.T., Ong D.E.H., Lim F.S.H., Teng K.W.W., McGovern N., Narayanan S., Ho W.Q., Cerny D., Tan H.K.K., Anicete R. (2016). A High-Dimensional Atlas of Human T Cell Diversity Reveals Tissue-Specific Trafficking and Cytokine Signatures. Immunity.

[49] Woon H.G., Braun A., Li J., Smith C., Edwards J., Sierro F., Feng C.G., Khanna R., Elliot M., Bell A.I. (2016). Compartmentalization of Total and Virus-Specific Tissue-Resident Memory CD8+ T Cells in Human Lymphoid Organs. PLOS Pathog..

[50] Swaims-Kohlmeier A., Haaland R.E., Haddad L.B., Sheth A.N., Evans-Strickfaden T., Lupo L.D., Cordes S., Aguirre A., Lupoli K., Chen C.-Y. (2016). Progesterone Levels Associate with a Novel Population of CCR5+CD38+ CD4 T Cells Resident in the Genital Mucosa with Lymphoid Trafficking Potential. J. Immunol..

[51] Smolders J., Heutinck K.M., Fransen N.L., Remmerswaal E.B.M., Hombrink P., Berge I.J.M.T., Van Lier R.A.W., Huitinga I., Hamann J. (2018). Tissue-resident memory T cells populate the human brain. Nat. Commun..

[52] Steinbach K., Vincenti I., Merkler D. (2018). Resident-Memory T Cells in Tissue-Restricted Immune Responses: For Better or Worse?. Front. Immunol..

[53] Schenkel J.M., Masopust D. (2014). Tissue-resident memory T cells. Immun..

[54] Klonowski K.D., Williams K.J., Marzo A.L.A., Blair D., Lingenheld E.G., Lefrançois L. (2004). Dynamics of Blood-Borne CD8 Memory T Cell Migration In Vivo. Immunity.

[55] Steinert E.M., Schenkel J.M., Fraser K.A., Beura L.K., Manlove L.S., Igyártó B.Z., Southern P.J., Masopust D. (2015). Quantifying Memory CD8 T Cells Reveals Regionalization of Immunosurveillance. Cell.

[56] Bromley S.K., Yan S., Tomura M., Kanagawa O., Luster A.D. (2012). Recirculating memory T cells are a unique subset of CD4+ T cells with a distinct phenotype and migratory pattern. J. Immunol..

[57] Ugur M., Schulz O., Menon M.B., Krueger A., Pabst O. (2014). Resident CD4+ T cells accumulate in lymphoid organs after prolonged antigen exposure. Nat. Commun..

[58] Muruganandah V., Sathkumara H.D., Navarro S., Kupz A. (2018). A Systematic Review: The Role of Resident Memory T Cells in Infectious Diseases and Their Relevance for Vaccine Development. Front. Immunol..

[59] Mullins D.W., Sheasley S.L., Ream R.M., Bullock T.N., Fu Y.-X., Engelhard V.H. (2003). Route of Immunization with Peptide-pulsed Dendritic Cells Controls the Distribution of Memory and Effector T Cells in Lymphoid Tissues and Determines the Pattern of Regional Tumor Control. J. Exp. Med..

[60] Sun Y.-Y., Peng S., Han L., Qiu J., Song L., Tsai Y., Yang B., Roden R.B., Trimble C.L., Hung C.-F. (2015). Local HPV Recombinant Vaccinia Boost Following Priming with an HPV DNA Vaccine Enhances Local HPV-Specific CD8+ T-cell-Mediated Tumor Control in the Genital Tract. Clin. Cancer Res..

[61] Morabito K.M., Ruckwardt T.R., Redwood A.J., Moin S.M., Price D.A., Graham B.S. (2016). Intranasal administration of RSV antigen-expressing MCMV elicits robust tissue-resident effector and effector memory CD8^+^ T cells in the lung. Mucosal Immunol..

[62] Çuburu N., Wang K., Goodman K.N., Pang Y.Y., Thompson C.D., Lowy D.R., Cohen J.I., Schiller J.T. (2014). Topical Herpes Simplex Virus 2 (HSV-2) Vaccination with Human Papillomavirus Vectors Expressing gB/gD Ectodomains Induces Genital-Tissue-Resident Memory CD8+T Cells and Reduces Genital Disease and Viral Shedding after HSV-2 Challenge. J. Virol..

[63] Calzascia T., Masson F., Di Berardino-Besson W., Contassot E., Wilmotte R., Aurrand-Lions M., Ruegg C., Dietrich P.-Y., Walker P.R. (2005). Homing Phenotypes of Tumor-Specific CD8 T Cells Are Predetermined at the Tumor Site by Crosspresenting APCs. Immunity.

[64] Çuburu N., Khan S., Thompson C.D., Kim R., Vellinga J., Zahn R., Lowy D.R., Scheper G., Schiller J.T. (2017). Adenovirus vector-based prime-boost vaccination via heterologous routes induces cervicovaginal CD8+ T cell responses against HPV16 oncoproteins. Int. J. Cancer.

[65] Sandoval F., Terme M., Nizard M., Badoual C., Bureau M.-F., Freyburger L., Clément O., Marcheteau E., Gey A., Fraisse G. (2013). Mucosal Imprinting of Vaccine-Induced CD8+ T Cells Is Crucial to Inhibit the Growth of Mucosal Tumors. Sci. Transl. Med..

[66] Maples P., Kumar P., Yü Y., Wang Z., Jay C., Pappen B., Rao D., Kuhn J., Nemunaitis J., Senzer N.N. (2010). FANG Vaccine: Autologous Tumor Cell Vaccine Genetically Modified to Express GM-CSF and Block Production of Furin. BioProcess J..

[67] Barve M., Kuhn J., Lamont J., Beitsch P., Manning L., Pappen B.O., Kumar P., Wallraven G., Senzer N.N., Nemunaitis J. (2016). Follow-up of bi-shRNA furin/GM-CSF Engineered Autologous Tumor Cell (EATC) Immunotherapy Vigil^®^ in patients with advanced melanoma. Biomed. Genet. Genom..

[68] Ghisoli M., Barve M., Schneider R., Mennel R., Lenarsky C., Wallraven G.O., Pappen B., LaNoue J., Kumar P., Nemunaitis D. (2015). Pilot Trial of FANG Immunotherapy in Ewing’s Sarcoma. Mol. Ther..

[69] Senzer N., Barve M., Kuhn J., Melnyk A., Beitsch P., Lazar M., Lifshitz S., Magee M., Oh J., Mill S.W. (2012). Phase I trial of “bi-shRNAi(furin)/GMCSF DNA/autologous tumor cell” vaccine (FANG) in advanced cancer. Mol. Ther..

[70] Senzer N., Barve M., Nemunaitis J., Kuhn J., Melnyk A., Beitsch P., Magee M., Oh J., Bedell C., Kumar P. (2013). Long Term Follow Up: Phase I Trial of “bi-shRNA furin/GMCSF DNA/Autologous Tumor Cell” Immunotherapy (FANG™) in Advanced Cancer. J. Vaccines Vaccine.

[71] Oh J., Barve M., Matthews C.M., Koon E.C., Heffernan T.P., Fine B., Grosen E., Bergman M.K., Fleming E.L., Demars L.R. (2016). Phase II study of Vigil^®^ DNA engineered immunotherapy as maintenance in advanced stage ovarian cancer. Gynecol. Oncol..

[72] Rocconi R.P., Grosen E.A., Ghamande S.A., Chan J.C.-K., Barve M.A., Oh J., Tewari D., Morris P.C., Stevens E.E., Bottsford-Miller J.N. Randomized Double-Blind Placebo Controlled Trial of Primary Maintenance Vigil Immunotherapy (VITAL study) in Stage III/IV Ovarian Cancer: Efficacy Assessment in BRCA1/2-wt Patients (Late Breaking Oral Presentation). Proceedings of the Society of Gynecologic Oncology Annual Meeting on Women’s Cancer.

[73] Herron J., Smith N., Stanbery L., Aaron P., Manning L., Bognar E., Wallraven G., Horvath S., Nemuanaitis J. (2020). Vigil: Personalized Immunotherapy Generating Systemic Cytotoxic T cell Response. Cancer Sci. Res..

[74] Perica K., Varela J.C., Oelke M., Schneck J. (2015). Adoptive T Cell Immunotherapy for Cancer. Rambam Maimonides Med. J..

[75] Wu T.-C., Xu K., Banchereau R., Marches F., Yu C.I., Martinek J., Anguiano E., Pedroza-Gonzalez A., Snipes G.J., O’Shaughnessy J. (2014). Reprogramming tumor-infiltrating dendritic cells for CD103+ CD8+ mucosal T-cell differentiation and breast cancer rejection. Cancer Immunol. Res..

[76] Grupp S.A., Kalos M., Barrett D., Aplenc R., Porter D.L., Rheingold S.R., Teachey D.T., Chew A., Hauck B., Wright J.F. (2013). Chimeric Antigen Receptor–Modified T Cells for Acute Lymphoid Leukemia. N. Engl. J. Med..

[77] Kochenderfer J.N., Dudley M.E., Kassim S.H., Somerville R.P., Carpenter R.O., Stetler-Stevenson M., Yang J.C., Phan G.Q., Hughes M.S., Sherry R.M. (2015). Chemotherapy-Refractory Diffuse Large B-Cell Lymphoma and Indolent B-Cell Malignancies Can Be Effectively Treated With Autologous T Cells Expressing an Anti-CD19 Chimeric Antigen Receptor. J. Clin. Oncol..

[78] Maude S.L., Frey N., Shaw P.A., Aplenc R., Barrett D.M., Bunin N.J., Chew A., Gonzalez V.E., Zheng Z., Lacey S.F. (2014). Chimeric antigen receptor T cells for sustained remissions in leukemia. N. Engl. J. Med..

[79] Porter D.L., Levine B.L., Kalos M., Bagg A., June C.H. (2011). Chimeric Antigen Receptor–Modified T Cells in Chronic Lymphoid Leukemia. N. Engl. J. Med..

[80] Schuster S.J., Svoboda J., Chong E.A., Nasta S.D., Mato A.R., Anak Ö., Brogdon J.L., Pruteanu-Malinici I., Bhoj V., Landsburg D. (2017). Chimeric Antigen Receptor T Cells in Refractory B-Cell Lymphomas. N. Engl. J. Med..

[81] Metzinger M.N., Verghese C., Hamouda D.M., Lenhard A., Choucair K., Senzer N., Brunicardi F.C., Dworkin L., Nemunaitis J. (2019). Chimeric Antigen Receptor T-Cell Therapy: Reach to Solid Tumor Experience. Oncology.

[82] Zhang H., Ye Z.-L., Yuan Z.-G., Luo Z.-Q., Jin H.-J., Qian Q.-J. (2016). New Strategies for the Treatment of Solid Tumors with CAR-T Cells. Int. J. Boil. Sci..

[83] Mackay L.K., Wynne-Jones E., Freestone D., Pellicci D.G., Mielke L.A., Newman D.M., Braun A., Masson F., Kallies A., Belz G.T. (2015). T-box Transcription Factors Combine with the Cytokines TGF-beta and IL-15 to Control Tissue-Resident Memory T Cell Fate. Immunity.

[84] Komdeur F.L., Wouters M.C.A., Workel H.H., Tijans A.M., Terwindt A.L., Brunekreeft K.L., Plat A., Klip H.G.A., Eggink F., Leffers N. (2016). CD103+ intraepithelial T cells in high-grade serous ovarian cancer are phenotypically diverse TCRαβ+ CD8αβ+ T cells that can be targeted for cancer immunotherapy. Oncotarget.

[85] Murray T., Marraco S.A.F., Baumgaertner P., Bordry N., Cagnon L., Donda A., Romero P., Verdeil G., Speiser D.E. (2016). Very Late Antigen-1 Marks Functional Tumor-Resident CD8 T Cells and Correlates with Survival of Melanoma Patients. Front. Immunol..

[86] Koh J., Kim S., Kim M.-Y., Go H., Jeon Y.K., Chung D.H. (2017). Prognostic implications of intratumoral CD103+ tumor-infiltrating lymphocytes in pulmonary squamous cell carcinoma. Oncotarget.

[87] Lohneis P., Sinn M., Bischoff S., Jühling A., Pelzer U., Wislocka L., Bahra M., Sinn B.V., Denkert C., Oettle H. (2017). Cytotoxic tumour-infiltrating T lymphocytes influence outcome in resected pancreatic ductal adenocarcinoma. Eur. J. Cancer.

[88] Yau C., Wolf D., Campbell M., Savas P., Lin S., Brown-Swigart L., Hirst G., Asare S., Zhu Z., Loi S. (2019). Abstract P3-10-06: Expression-based immune signatures as predictors of neoadjuvant targeted-/chemo-therapy response: Experience from the I-SPY 2 TRIAL of 1000 patients across 10 therapies. Poster Sess. Abstr..

[89] Adams S., Schmid P., Rugo H., Winer E., Loirat D., Awada A., Cescon D., Iwata H., Campone M., Nanda R. (2019). Pembrolizumab monotherapy for previously treated metastatic triple-negative breast cancer: Cohort A of the phase II KEYNOTE-086 study. Ann. Oncol..

[90] Adams S., Loi S.M., Toppmeyer D., Cescon D., De Laurentiis M., Nanda R., Winer E., Mukai H., Tamura K., Armstrong A. (2019). Pembrolizumab monotherapy for previously untreated, PD-L1-positive, metastatic triple-negative breast cancer: Cohort B of the phase II KEYNOTE-086 study. Ann. Oncol..

[91] Loi S., Schmid P., Cortés J., Cescon D.W., Winer E.P., Toppmeyer D., Rugo H.S., de Laurentiis M., Nanda R., Iwata H. (2019). RNA molecular signatures as predictive biomarkers of response to monotherapy pembrolizumab in patients with metastatic triple negative breast cancer: KEYNOTE-086. Cancer Res..

[92] Choucair K., Morand S., Stanbery L., Edelman G., Dworkin L., Nemunaitis J. (2020). TMB: A promising immune-response biomarker, and potential spearhead in advancing targeted therapy trials. Cancer Gene Ther..

[93] Disis M.L., Taylor M.H., Kelly K., Beck J.T., Gordon M., Moore K.M., Patel M.R., Chaves J., Park H., Mita A.C. (2019). Efficacy and Safety of Avelumab for Patients With Recurrent or Refractory Ovarian Cancer. JAMA Oncol..

[94] Pujade-Lauraine E., Fujiwara K., Ledermann J., Oza A., Kristeleit R., Ray-Coquard I., Richardson G.E., Sessa C., Yonemori K., Banerjee S. Avelumab alone or in combination with pegylted liposomal doxorubicin versus pegylated liposomal doxorubicin alone in platinum-resistant or refractory epithelial ovarian cancer: Primary and biomarker analysis of hte phase III JAVELIN Ovarian 200 trial. Proceedings of the Society of Gynecologic Oncology Annual Meeting.

[95] Eskander R.N., Ledermann J.A., Birrer M.J., Fujiwara K., Gaillard S., Richardson G.E., Wei C., Baig M.A., Zohren F., Monk B.J. (2019). JAVELIN ovarian PARP 100 study design: Phase III trial of avelumab + chemotherapy followed by avelumab + talazoparib maintenance in previously untreated epithelial ovarian cancer. J. Clin. Oncol..

[96] Hodi F.S., O’Day S.J., McDermott D.F., Weber R.W., Sosman J.A., Haanen J.B., Gonzalez R., Robert C., Schadendorf D., Hassel J.C. (2010). Improved Survival with Ipilimumab in Patients with Metastatic Melanoma. N. Engl. J. Med..

[97] Wada S., Jackson C.M., Yoshimura K., Yen H.-R., Getnet D., Harris T.J., Goldberg M.V., Bruno T.C., Grosso J.F., Durham N. (2013). Sequencing CTLA-4 blockade with cell-based immunotherapy for prostate cancer. J. Transl. Med..

[98] Loibl S., Untch M., Burchardi N., Huober J., Sinn B., Blohmer J.-U., Grischke E.-M., Furlanetto J., Tesch H., Hanusch C. (2019). A randomised phase II study investigating durvalumab in addition to an anthracycline taxane-based neoadjuvant therapy in early triple-negative breast cancer: Clinical results and biomarker analysis of GeparNuevo study. Ann. Oncol..

